# Effects of altitude and sociodemographic factors on cardiometabolic disorders in the southwestern plateau areas of China

**DOI:** 10.3389/fcvm.2026.1709651

**Published:** 2026-05-15

**Authors:** Nan Zhang, Wenlong Zhu, Yu Xia, Lin Duo, Zhiling Luo, Mingjing Tang, Da Zhu

**Affiliations:** 1 Fuwai Yunnan Hospital, Chinese Academy of Medical Sciences, Affiliated Cardiovascular Hospital of Kunming Medical University, Kunming, China; 2 Yunnan Provincial Cardiovascular Clinical Medical Research Center, Kunming, China

**Keywords:** altitude, diabetes, dyslipidemia, hypertension, plateau areas

## Abstract

**Background:**

Cardiometabolic disorders, including hypertension, dyslipidemia, and diabetes, are increasingly prevalent globally, yet evidence in economically underdeveloped high-altitude areas remains limited. This study sought to assess the prevalence and control of these diseases in the southwestern plateau areas of China and to evaluate their associations with altitude and sociodemographic factors.

**Methods:**

We conducted two cross-sectional surveys among adults aged ≥35 years in the southwestern plateau areas of China. Disease definitions were based on measured biomarkers, self-reported diagnoses, and medication use. Logistic regression and restricted cubic spline models were applied to examine associations between sociodemographic factors, altitude, and prevalence and control rates of cardiometabolic disorders.

**Results:**

7,668 adults with an average age of 54.88 ± 12.64 years were investigated. The prevalence, awareness, treatment, and control rates were 46.20%, 58.65%, 43.80%, and 15.75% for hypertension; 37.06%, 15.90%, 5.77%, and 2.11% for dyslipidemia; 11.79%, 27.54%, 21.68%, and 6.86% for diabetes. Higher disease prevalence was associated with male, older age, higher income, overweight/obesity, and alcohol use. Reduced control and treatment rates were observed at altitudes ≥2,242 m, among younger adults, smokers, and drinkers. Altitude significantly modified the association between sociodemographic factors and cardiometabolic disorders. Furthermore, there was a non-linear relationship between these rates and both age and altitude of residence.

**Conclusions:**

This study reveals a high burden of cardiometabolic disorders with suboptimal management in the southwestern plateau areas of China. Altitude and sociodemographic factors significantly interact with disease prevalence and control. Targeted public health efforts should prioritize high-risk populations and regions to improve prevention and management.

## Introduction

Globally, cardiovascular disease (CVD) is the leading cause of death, accounting for 30% of all deaths (1). In 2021, the global prevalence of CVD increased to 7.85%, with the prevalence in China even higher at 9.58% (2). Hypertension, dyslipidemia, and diabetes are major cardiometabolic disorders and risk factors for CVD. Hypertension causes over 100,000 deaths worldwide each year and is the leading preventable risk factor for CVD and all-cause mortality (3). Dyslipidemia is the second most important but often overlooked risk factor for CVD (4). Additionally, diabetes is strongly associated with CVD and is an independent risk factor for both incident and fatal ischemic heart disease and stroke (5).

The global prevalence of cardiometabolic disorders, including hypertension, dyslipidemia, and diabetes, is increasing, accompanied by suboptimal awareness, treatment, and control rates (6–9). In China, similar challenges exist amid significant regional heterogeneity. National data indicate that hypertension prevalence among adults aged 18–69 rose from 20.8% in 2004 to 29.6% in 2010, then declined to 24.7% in 2018 (7). Regional studies, such as one conducted in five southwestern provinces (2016–2018), reported a hypertension prevalence of 40.3%, with awareness, treatment, and control rates of 43.7%, 29.8%, and 9.6%, respectively (8). For dyslipidemia, a large cross-sectional survey (2014–2019) found a prevalence of 33.8% and a treatment rate of only 14.1% in China (9). Diabetes prevalence has risen dramatically from less than 1% in the 1980s to 12.4% in 2018, with awareness, treatment, and control rates at 36.7%, 32.9%, and 50.1%, respectively (10, 11). Although these disease burdens are increasingly documented in developed regions, economically underdeveloped areas, particularly those with unique environmental and sociocultural characteristics, remain understudied (12, 13).

The prevalence, awareness, treatment, and control of hypertension, diabetes, and dyslipidemia vary in different populations. Factors such as age, altitude of residence, sex, education, socioeconomic status, and behavior may contribute to these variations (14–19). Older age, along with higher education and income, is often associated with higher disease risk but also better awareness and control (14, 18, 19). Additionally, evidence regarding altitude is inconsistent: some studies suggest high altitude increases hypertension and diabetes risk, while others indicate a protective effect against hypertension and dyslipidemia (15–17). Nonetheless, few studies have systematically examined the joint effects of altitude and sociodemographic factors on disease control.

Yunnan Province is situated in the southwestern plateau region of China and remains an economically underdeveloped area, with a per capita GDP of 65300 CNY in 2023, which is only 71% of the national average (20). The province exhibits exceptional geographic diversity, with altitudes ranging from 76 to 6740 meters (21). Additionally, Yunnan is characterized by a multi-ethnic population and distinctive dietary habits, including high sodium intake, low calcium consumption, and prevalent consumption of pickled vegetables (22). These factors, along with its unique geography and climate, may collectively influence the incidence and progression of cardiometabolic diseases. Nonetheless, few studies have examined the effects of altitude and sociodemographic characteristics on the prevalence and control of cardiometabolic disorders in this region.

In this study, we conducted two cross-sectional surveys among adults aged ≥35 years to assess the prevalence, awareness, treatment, and control of hypertension, dyslipidemia, and diabetes in the southwestern plateau areas of China. Furthermore, we evaluated the effects of altitude and sociodemographic factors on these outcomes, providing essential evidence for targeted public health policy.

## Methods

### Study design

The data for this study were from two cross-sectional studies with no overlap in the study populations. The first study was the “Cardiovascular Disease and Risk Factor Surveillance Programme for Chinese Residents”, conducted in Yunnan Province from January to December 2021. This program focused on screening for CVDs and their risk factors among permanent residents aged 18 years and older. A stratified multi-stage random sampling method was used to select 16 townships across 8 districts and counties in Yunnan Province, with a median altitude of 1,896 meters. Based on the sampling procedure and the prevalence of hypertension in Yunnan Province (38.5%) (23), we estimated that 9,600 participants would be necessary for the program. The project has been approved by the Ethics Committee of Fuwai Hospital/National Center for Cardiovascular Disease (Grant number: 2020–1360). The second study was a survey conducted from September 2023 to January 2024, aimed at investigating CVD risk factors among residents of high-plateau areas in Yunnan Province. A multi-stage stratified cluster sampling method was used to select participants from 16 townships across 2 districts and counties, where the median altitude is 3,001 meters. Based on the sampling procedure and the prevalence of hypertension in Yunnan Province, we estimated that 2,560 participants would be necessary for the study. This survey was approved by the Ethics Committee of Fuwai Yunnan Hospital, Chinese Academy of Medical Sciences (Grant number: 2023–026-01). Details regarding the sampling procedures and data collection for both studies have been described in a previous study (24). Additionally, comprehensive information about sampling methodology and on-site screening protocols (Supplementary Figure S1) can be found in the “Study Design and Participants” section of the Supplementary Materials.

**Figure 1:**
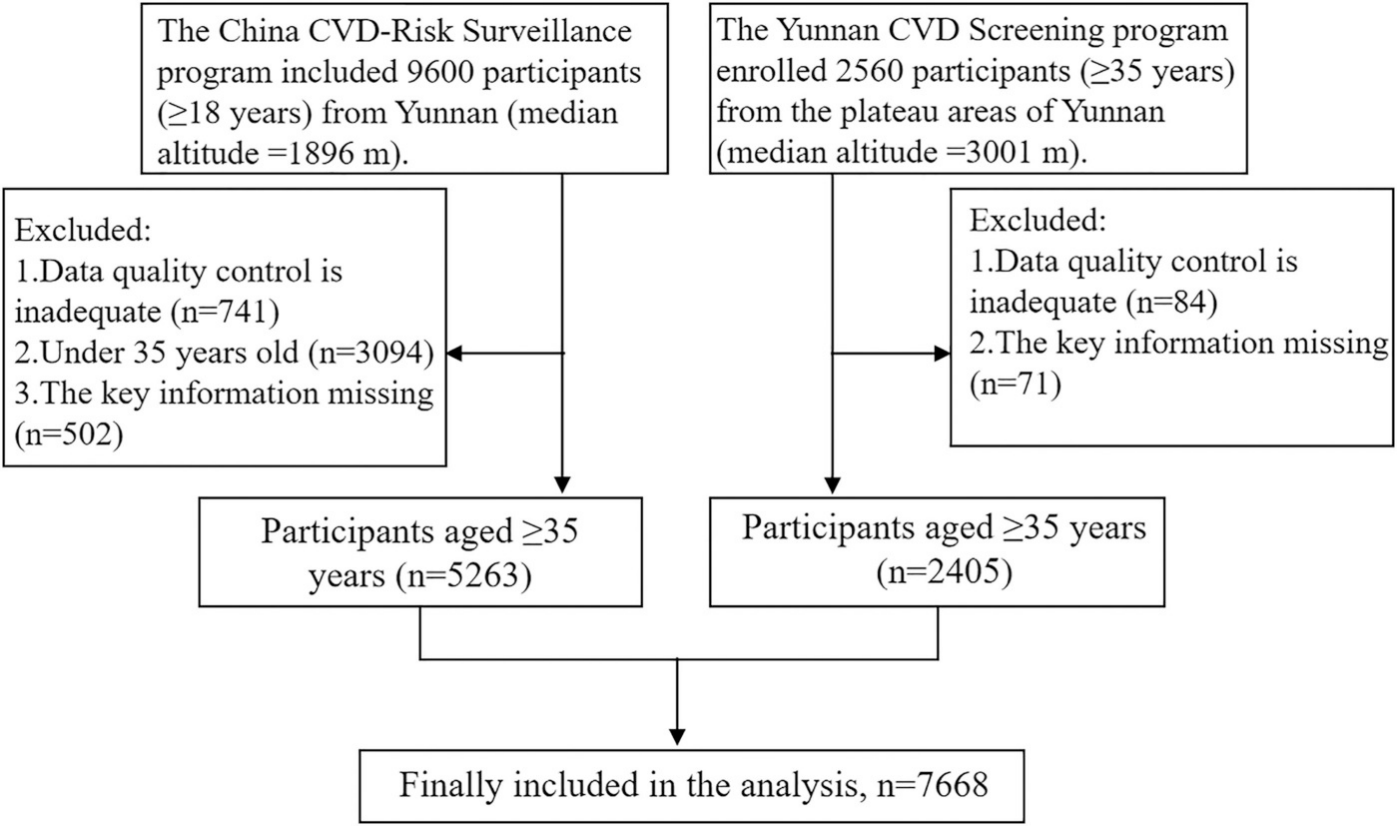
Flowchart of participants inclusion.

The two studies were combined into one dataset. As the prevalence of cardiometabolic disorders among individuals <35 years is very low, to ensure comparability with other studies, permanent residents aged 35 and above were included. Participants with missing data regarding key physiological parameters (e.g., blood pressure and fasting glucose) defining the diseases were excluded, resulting in a total of 7,668 participants over the age of 35 included in the study. The sample inclusion process is shown in Figure 1. The study adhered to the Strengthening the Reporting of Observational Studies in Epidemiology (STROBE) guidelines (25).

### Data collection

Both cross-sectional studies used the same survey protocols and data collection schedules, and the surveys were conducted at the township/community health center. A team of approximately 20 trained professionals, including physicians, sonographers, nurses, pharmacists, and medical students, carried out the surveys. The questionnaires used in the two studies were identical to those utilized by Li et al. (26), which collected information on individuals' sociodemographic status, education level, lifestyle behaviors, major medical history, and medication use (such as hypoglycemic, hypertensive, and lipid-lowering medications). Fasting blood samples were collected in the morning after at least 8 h of fasting and used to measure fasting plasma glucose (FPG), total cholesterol (TC), triglycerides (TG), high-density lipoprotein cholesterol (HDL-C), and low-density lipoprotein cholesterol (LDL-C).

### Definitions of prevalence, awareness, treatment, and control of the three diseases

Hypertension was defined as systolic blood pressure ≥140 mmHg, diastolic blood pressure ≥ 90 mmHg, or self-reported antihypertensive use (27). Dyslipidemia was characterized by self-reported history or TC ≥ 6.22 mmol/L, TG ≥ 2.26 mmol/L, HDL-C < 1.04 mmol/L, or LDL-C ≥ 4.14 mmol/L (28). Diabetes was identified by FPG ≥ 7.0 mmol/L or self-reported medication/insulin use (29). Awareness refers to patients who self-report having been diagnosed with these conditions by a physician. Treatment is defined based on patients’ self-reported medication usage. Control indicates those undergoing treatment whose relevant indicators meet control standards (27, 29).

Current smoking was defined as regular consumption of ≥7 cigarettes weekly sustained for at least 6 months (30). Current alcohol drinking was classified based on any beverage consumption occurring within the past month (31). Body mass index (BMI) was calculated as weight(kg)/(height(m))^2^. According to Chinese criteria of weight for adults, participants were divided into underweight (BMI < 18.5 kg/m^2^), normal weight (18.5 to <24 kg/m^2^), overweight (24 to < 28 kg/m^2^), and obese (≥ 28 kg/m^2^) (32).

Per capita annual household income was defined as the average income of family members living together for the long term in the previous year. According to the 2019 national quintile income groups in China, the per capita disposable income was 7,380 yuan in the low-income group, 15,777 yuan in the lower-middle-income group, and 25,035 yuan in the middle-income group. The per capita disposable income in Yunnan Province in 2019 was 22,082 yuan. To align with both national standards and local economic conditions, we set a cutoff of 21,000 yuan. Individuals with a per capita income <21,000 yuan were classified into the low-to-middle income group.

### Altitude assessment

In China, township governments are generally located in areas with convenient transportation and serve as the main residential and activity centers for most residents. Therefore, the altitude data of township governments provided by the Yunnan Provincial Map Research Institute was used to represent the typical residential altitude of participants (24, 33). Based on the altitude distribution of the study population, residential altitude was categorized into three groups by tertiles, ensuring relatively balanced sample sizes across groups and capturing meaningful exposure gradients: Altitude 1: 818–1,895 m, Altitude 2: 1,896–2,241 m, Altitude 3: ≥2,242 m. If the number of outcome events (awareness, treatment, and control) in Altitude 3 was less than 50, this group was merged with Altitude 2 (i.e., ≥1,896 m) to ensure statistical stability.

### Statistical analysis

For continuous variables, those with a normal distribution were presented using the mean and standard deviation (SD) and compared between groups using the *t*-test; those with a non-normal distribution were presented using the median, 25th percentile (P25), and 75th percentile (P75), with comparisons between groups made using the Mann–Whitney *U*-test. Categorical variables were described using frequencies and percentages, with group comparisons conducted using the Chi-square test or Fisher's exact test. Logistic regression models were used to calculate odds ratios (ORs) and 95% confidence intervals (95% CIs) to estimate the association between sociodemographic characteristics, altitude of residence, and the prevalence, awareness, treatment, and control of each disease. Subgroup analyses and interaction analyses were performed to explore the interaction between altitude and demographic characteristics in the prevalence, awareness, treatment, and control of different diseases. Restricted cubic spline (RCS) regression models with four knots (at the 5th, 35th, 65th, and 95th percentiles) were used to investigate non-linear associations between age, altitude of residence, and disease prevalence, awareness, treatment, and control. According to the Bonferroni correction method, *p* for nonlinearity ≤ 0.004 (= 0.05/12) was considered statistically significant. Multivariate logistic regression, subgroup analyses, interaction analyses, and RCS regression models were adjusted for sex, age, education status, income, overweight/obesity, current smoking, and current alcohol drinking status. IBM SPSS 26.0 statistical software and R 4.4.1 software (https://www.r-project.org/) were used for the statistical analysis. All statistical tests were two-tailed, and *p*-values < 0.05 were regarded as statistically significant.

## Results

### General characteristics of the study population

The study included 7,668 participants aged 35 years and older, with a mean age of 54.88 ± 12.64 years and a median altitude of residence of 1942 (P25, P75: 1880, 2864) meters. Of all participants, 47.18% were male, 56.89% had an education level at primary school or below, 59.18% reported a per capita disposable income <21,000 yuan, 27.71% were current smokers, 19.20% were current alcohol drinkers, and 48.60% were overweight/obese. Except for age, TC and LDL-C, other characteristics differed significantly between males and females (*p* < 0.05, Table 1).

**Table 1:** Basic characteristics of participants by Sex groups*.

Characteristics	Overall	Female	Male	*p*-value
N	7,668	4,050 (52.82)	3,618 (47.18)	-
Altitude, meters	1,942 (1,880, 2,864)	1,942 (1,880, 2,864)	1,942 (1860, 2864)	<0.001^†^
Altitude groups, meters				
Altitude 1: [818, 1,896)	2,800 (36.52)	1,386 (34.22)	1,414 (39.08)	<0.001
Altitude 2: [1,896, 2,242)	2,462 (32.11)	1,295 (31.98)	1,167 (32.26)	
Altitude 3: ≥2,242	2,406 (31.38)	1,369 (33.80)	1,037 (28.66)	
Age, years	54.88 ± 12.64	54.77 ± 12.56	55.02 ± 12.72	0.387
Age groups, years				
<60	4,050 (52.82)	2,755 (68.02)	2,402 (66.39)	0.128
≥60	3,618 (47.18)	1,295 (31.98)	1,216 (33.61)	
Educational status				
Primary school and below	4,362 (56.89)	2,665 (65.80)	1,697 (46.90)	<0.001
Junior high school and above	3,306 (43.11)	1,385 (34.20)	1,921 (53.10)	
Income, yuan				
<21,000	4,538 (59.18)	2,483 (61.31)	2,055 (56.8)	<0.001
≥21,000	3,130 (40.82)	1,567 (38.69)	1,563 (43.20)	
Current smoking				
No	5,543 (72.29)	3,994 (98.62)	1,549 (42.81)	<0.001
Yes	2,125 (27.71)	56 (1.38)	2,069 (57.19)	
Current alcohol drinking				
No	6,196 (80.80)	3,875 (95.68)	2,321 (64.15)	<0.001
Yes	1,472 (19.20)	175 (4.32)	1,297 (35.85)	
Overweight/obesity				
No	3,941 (51.40)	2,157 (53.26)	1,784 (49.31)	0.001
Yes	3,727 (48.60)	1,893 (46.74)	1,834 (50.69）	
BMI, kg/m^2^	24.09 ± 3.64	23.99 ± 3.71	24.21 ± 3.56	0.010
DBP, mmHg	81.00 (73.33, 88.67)	78.67 (71.67, 86.33)	83.00 (76.00, 91.00)	<0.001^†^
SBP, mmHg	130.33 (118.33, 145.33)	128.50 (116.33, 144.67)	132.33 (120.33, 146.00)	<0.001^†^
FBG, mmol/L	5.06 (4.52, 5.71)	4.97 (4.47, 5.60)	5.14 (4.60, 5.83)	<0.001^†^
TC, mmol/L	4.99 ± 1.10	4.97 ± 1.09	5.00 ± 1.11	0.231
TG, mmol/L	1.26 (0.87, 1.90)	1.19 (0.84, 1.73)	1.35 (0.90, 2.16)	<0.001^†^
LDL-C, mmol/L	2.91 ± 0.86	2.90 ± 0.85	2.91 ± 0.87	0.590
HDL-C, mmol/L	1.47 ± 0.39	1.52 ± 0.38	1.42 ± 0.39	<0.001

* Numbers are given as *n* (%), median (P25, P75) or mean ± standard deviation (SD).

† Differences among the two groups were compared using the Mann–Whitney *U*-test.

Relationship between sociodemographic characteristics and prevalence, awareness, treatment, and control of hypertension, dyslipidemia, and diabetes.

### Hypertension

**Figure 2:**
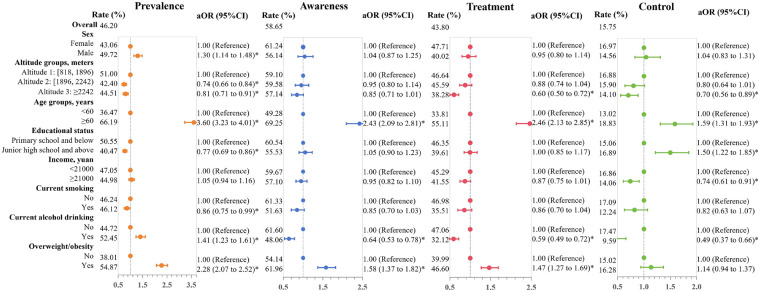
Forest plot of hypertension prevalence, awareness, treatment, and control. The estimated adjusted odds ratios (aORs) are indicated by points, while the 95% confidence intervals (95% CIs) for each regression coefficient are illustrated by line segments. Each outcome variable was estimated in an independent model, the models were adjusted for sex, age, education status, income, overweight/obesity, current smoking, and alcohol drinking.

Among all participants, the prevalence of hypertension was 46.20%, and the rates of awareness, treatment, and control were 58.65%, 43.80%, and 15.75%, respectively (Figure 2). Univariable logistic regression models revealed a higher hypertension prevalence among males, those living at lower altitudes, older age groups, those with lower educational attainment, individuals who were overweight/obese, and those who drank alcohol (*p* < 0.05). Among hypertensive patients, increased rates of disease awareness, treatment, and control were significantly associated with being female, older, non-smokers, and not drinking (*p* < 0.05, Supplementary Table S1).

Multivariable logistic regression models demonstrated substantial concordance with univariable findings. Specifically, males exhibited a significantly higher hypertension prevalence compared to females (aOR=1.30, 95% CI: 1.14 to 1.48). Residents living at altitudes above 2,242 meters demonstrated negative associations for hypertension prevalence (aOR=0.81, 95% CI: 0.71 to 0.91), along with lower rates of hypertension treatment (aOR=0.60, 95% CI: 0.50 to 0.72) and control (aOR=0.70, 95% CI: 0.56 to 0.89) compared to the reference group (818–1,896 meters). Participants aged ≥60 years manifested substantially higher rates of hypertension prevalence (aOR=3.60, 95% CI: 3.23 to 4.01), awareness (aOR=2.43, 95% CI: 2.09 to 2.81), treatment (aOR=2.46, 95% CI: 2.13 to 2.85), and control (aOR=1.59, 95% CI: 1.31 to 1.93) in comparison to the younger group. Higher educational attainment was correlated with a decreased prevalence of hypertension (aOR=0.77, 95% CI: 0.69 to 0.86) and improved control rates (aOR=1.50, 95% CI: 1.22 to 1.85). While household income showed no significant associations with hypertension prevalence, awareness, or treatment, blood pressure control was found to be poorer among individuals with higher incomes (aOR=0.74, 95% CI: 0.61 to 0.91). In addition, individuals who were overweight or obese and engaged in alcohol consumption had a higher prevalence of hypertension and lower rates of awareness, treatment, and control (Figure 2).

The associations between the prevalence of hypertension and demographic and behavioral factors (sex, age, educational attainment, current smoking, and alcohol consumption) were affected by the altitude of residence (*p* for interaction <0.05). Interaction effects concerning hypertension awareness were observed between altitude and both sex and education level (*p* for interaction <0.05). Regarding treatment, altitude showed interaction effects with age and educational attainment (*p* for interaction <0.05, Supplementary Table S2).

### Dyslipidemia

**Figure 3:**
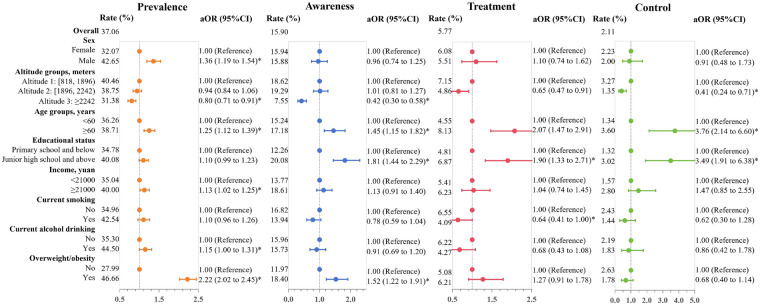
Forest plot of dyslipidemia prevalence, awareness, treatment, and control. The estimated adjusted odds ratios (aORs) are indicated by points, while the 95% confidence intervals (95% CIs) for each regression coefficient are illustrated by line segments. Each outcome variable was estimated in an independent model, the models were adjusted for sex, age, education status, income, overweight/obesity, current smoking, and alcohol drinking. In the analysis of dyslipidemia treatment and control rates, Altitude 2 and 3 were merged into Altitude 2 (≥1,896 meters).

The prevalence of dyslipidemia was 37.06%. Among the individuals with dyslipidemia, 15.90% were aware of their condition, 5.77% were receiving treatment, and only 2.11% had blood lipids under control (Figure 3). Univariable logistic regression analyses indicated a higher prevalence of dyslipidemia among males, individuals residing at altitudes below 2,242 meters, those aged 60 years and older, individuals with a high school or above education, those with higher income levels, current smokers, alcohol drinkers, and those who were overweight/obese. Among patients with dyslipidemia, disease awareness, treatment, and control showed positive associations with lower altitude of residence and advanced educational attainment (*p* < 0.05, Supplementary Table S3).

Multivariate logistic regression models revealed a higher prevalence of dyslipidemia in males compared to females (aOR=1.36, 95% CI: 1.19 to 1.54). Compared to those residing below 1,896 meters, individuals lived at altitudes above 2,242 meters had a lower prevalence of dyslipidemia (aOR=0.80, 95% CI: 0.71 to 0.91) and a lower rate of dyslipidemia awareness (aOR=0.42, 95% CI: 0.30 to 0.58); individuals lived above 1,896 meters had lower rates of dyslipidemia treatment (aOR=0.65, 95% CI: 0.47 to 0.91) and control (aOR=0.41, 95% CI: 0.24 to 0.71). Notably, participants aged ≥60 years exhibited elevated dyslipidemia prevalence (aOR=1.25, 95% CI: 1.12 to 1.39), awareness rate (aOR=1.45, 95% CI: 1.15 to 1.82), treatment engagement (aOR=2.07, 95% CI: 1.47 to 2.91) and control efficacy (aOR=3.76, 95% CI: 2.14 to 6.60) compared to the younger group. Higher-educated groups showed significant increases in the rates of dyslipidemia awareness (aOR=1.81, 95% CI: 1.44 to 2.29), treatment (aOR=1.90, 95% CI: 1.33 to 2.71), and control (aOR=3.49, 95% CI: 1.91 to 6.38). Compared with those with household income < 21,000 yuan, dyslipidemia prevalence rates were significantly elevated in higher-income groups (aOR=1.13, 95% CI: 1.02 to 1.25), while no significant differences emerged in dyslipidemia awareness, treatment, or control rates. In addition, current alcohol drinkers had a higher prevalence of dyslipidemia (aOR=1.15, 95% CI: 1.00 to 1.31), and smokers had a lower rate of lipid therapy (aOR=0.64, 95% CI: 0.41 to 1.00). The rates of prevalence (aOR=2.22, 95% CI: 2.02 to 2.45) and awareness (aOR=1.52, 95% CI: 1.22 to 1.91) of dyslipidemia were higher among overweight or obese individuals (Figure 3).

Altitude of residence exhibits multidimensional interactions with sociodemographic and metabolic factors across dyslipidemia outcomes. In terms of prevalence, altitude demonstrated interaction effects with educational attainment, income level, and overweight/obesity. Regarding dyslipidemia awareness, altitude showed interaction effects with age, educational attainment, and overweight/obesity. In dyslipidemia treatment, altitude interacted specifically with age and overweight/obesity; in dyslipidemia control, altitude exhibited interaction effects with educational attainment (*p* for interaction <0.05) (Supplementary Table S4).

### Diabetes

**Figure 4:**
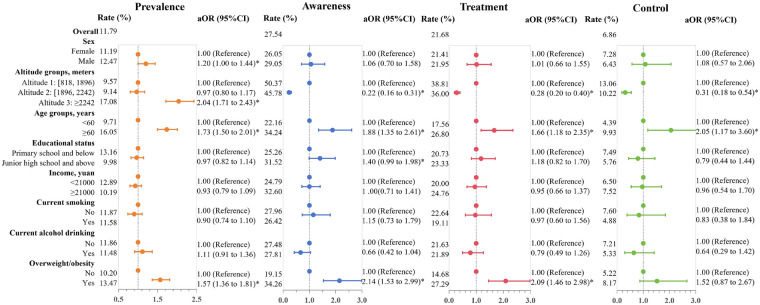
Forest plot of diabetes prevalence, awareness, treatment, and control. The estimated adjusted odds ratios (aORs) are indicated by points, while the 95% confidence intervals (95% CIs) for each regression coefficient are illustrated by line segments. Each outcome variable was estimated in an independent model, the models were adjusted for sex, age, education status, income, overweight/obesity, current smoking, and alcohol drinking. In the analysis of diabetes awareness, treatment, and control, Altitude 2 and 3 were merged into Altitude 2 (≥1,896 meters).

The overall prevalence of diabetes was 11.79%. Among individuals with diabetes, 27.54% were diagnosed, 21.68% were treated, and 6.86% had their glycaemia controlled (Figure 4). Univariate logistic regression identified significantly increased prevalence of diabetes among those living at altitudes ≥2,242 meters, individuals aged ≥60 years, and those with lower educational attainment, lower income, and overweight/obesity. Among those with diabetes who lived below 1,896 meters and were older, there were increased rates of diabetes awareness, treatment, and control (*p* < 0.05, Supplementary Table S5).

Multivariate logistic regression showed that the prevalence of diabetes was significantly higher in males (aOR=1.20, 95% CI: 1.00 to 1.44). Individuals residing at higher altitudes (≥1,896 meters) had significantly lower rates for diabetes awareness (aOR=0.22, 95% CI: 0.16 to 0.31), treatment (aOR=0.28, 95% CI: 0.20 to 0.40), and control (aOR=0.31, 95% CI: 0.18 to 0.54) compared to those living at lower altitudes. Individuals aged 60 years and above had the increased rates of diabetes prevalence (aOR=1.73, 95% CI: 1.50 to 2.01), awareness (aOR=1.88, 95% CI: 1.35 to 2.61), treatment (aOR=1.66, 95% CI: 1.18 to 2.35), and control (aOR=2.05, 95% CI: 1.17 to 3.60). Individuals with high school or higher education showed an increased diabetes awareness rate (aOR=1.40, 95% CI: 0.99 to 1.98). Overweight/obesity was associated with an increased prevalence of diabetes (aOR=1.57, 95% CI: 1.36 to 1.81) and higher rates of awareness (aOR=2.14, 95% CI:1.53 to 2.99) and treatment (aOR=2.09, 95% CI:1.46 to 2.98) (Figure 4). Interaction analyses demonstrated significant interaction effects between altitude and age, socioeconomic status, and overweight/obesity in diabetes prevalence. Furthermore, regarding diabetes awareness and treatment, altitude showed interaction effects with educational attainment and overweight/obesity (*p* for interaction <0.05) (Supplementary Table S6**)**.

### Nonlinear relationship between age and altitude of residence with prevalence, awareness, treatment, and control of hypertension, dyslipidemia, and diabetes

**Figure 5:**
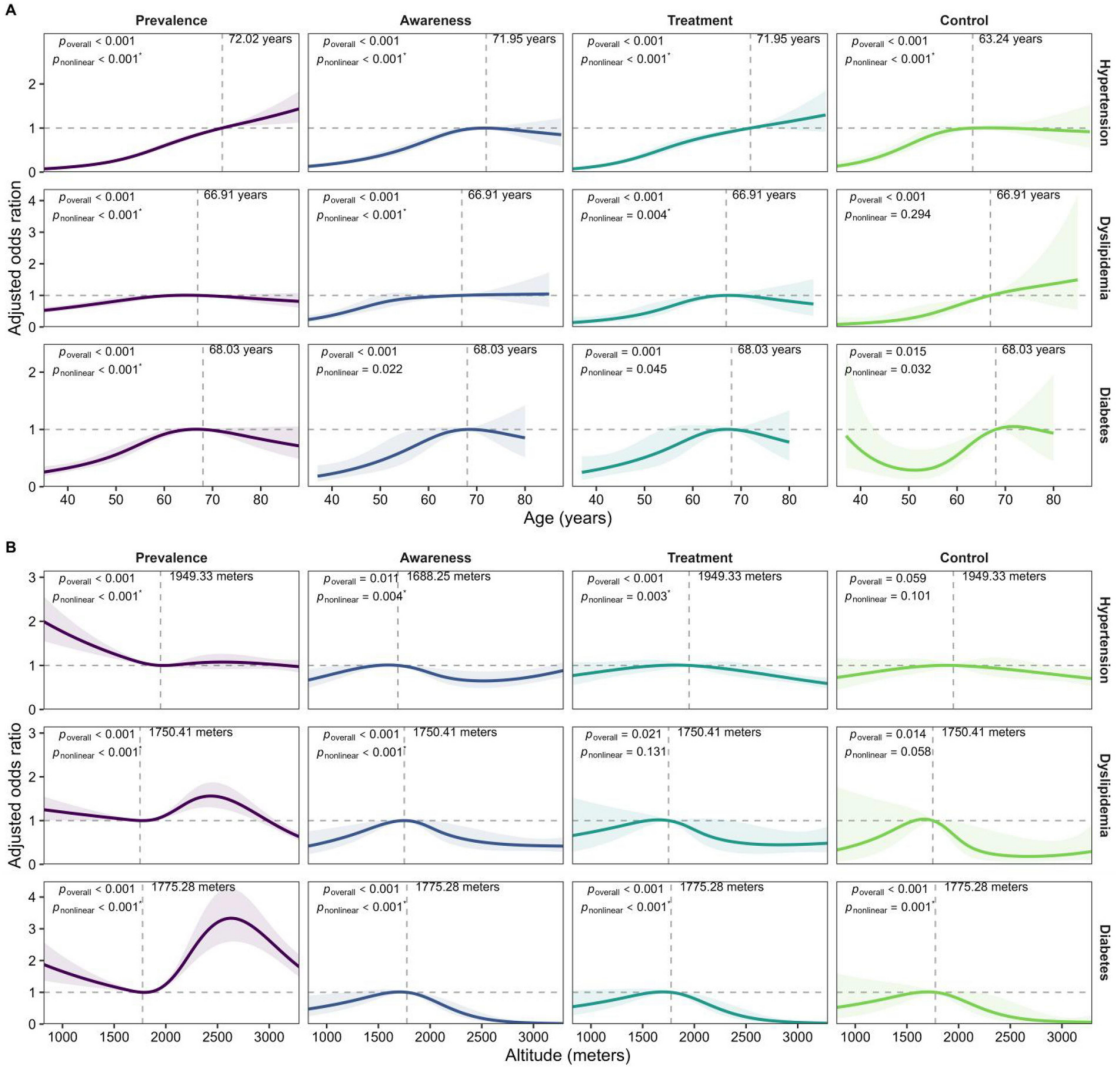
Nonlinear relationship between age **(A)**, altitude of residence **(B)**, and the rates of prevalence, awareness, treatment, and control of hypertension, dyslipidemia, and diabetes. The analysis included four nodes, corresponding to the 5th, 35th, 65th, and 95th percentiles. Each outcome variable was estimated in an independent model, the spline models adjusted for sex, education status, income, current smoking and alcohol drinking, and overweight/obesity. The solid lines represent adjusted odds ratios, the shaded areas indicate the 95% confidence intervals. The horizontal dashed gray line represents an adjusted odds ratio of 1. The vertical dashed gray line, along with the text, indicates the age/altitude of the reference group.* denotes statistical significance compared with the multiple testing threshold (*α*'=0.004).

The prevalence, awareness, and treatment rates of hypertension, dyslipidemia, and diabetes demonstrated significant non-linear relationships with age (*p* for nonlinear < 0.001). Specifically, hypertension prevalence, awareness, treatment, and control rates exhibited an increasing trend in individuals aged ≥35 years, while stabilization of awareness and control rates occurred in those aged ≥ 72 years and ≥ 63 years, respectively (*p* for nonlinear < 0.001). Similarly, the rates of dyslipidemia prevalence, awareness, and treatment also increased non-linearly with age among people younger than 67 years, but these rates began to stabilize, and even decreased for those aged over 67 years (*p* for nonlinear < 0.05). For individuals younger than 68 years, the rates of diabetes prevalence, awareness, and treatment increased nonlinearly with age, with control rates displaying an initial decline (the nadir at 51 years old) followed by recovery. Conversely, for individuals ≥68 years, these rates decreased with age (*p* for nonlinear <0.05, Figure 5A).

The rates of prevalence and awareness of hypertension, dyslipidemia, and diabetes exhibited a non-linear relationship with altitude of residence (*p* for nonlinear < 0.001). Specifically, among individuals living below 1,949 meters, the prevalence of hypertension demonstrated a nonlinear decreasing trend, while the treatment rates showed a nonlinear increasing trend with ascending altitude. In populations living above 1,948 meters, both the prevalence and treatment rates of hypertension initially stabilized before declining with increasing altitude. The awareness of hypertension increased nonlinearly with the elevation of altitude until reaching 1,688 meters, followed by an initial decrease (with the lowest point at 2,521 meters) before increasing once again (*p* for nonlinear < 0.05). The prevalence of dyslipidemia decreased with higher altitude among populations living below 1,750 meters, while it increased in those living above 1,750 meters, peaking at 2,434 meters before subsequently declining. Dyslipidemia awareness rate increased nonlinearly with altitude for those living below 1,750 meters, whereas in those above this altitude, awareness rate tended to decrease before stabilizing with increasing altitude (*p* for nonlinear *<* 0.001). For individuals residing at altitudes below 1,775 meters, the prevalence of diabetes decreased, while the rates of diabetes awareness, treatment, and control improved with increasing altitude. In contrast, for those at altitude above 1,775 meters, the prevalence of diabetes tended to rise (peaking at 2,633 meters) before decreasing, and the rates of awareness, treatment, and control declined as altitude increased (*p* for nonlinear <0.001, Figure 5B). According to the Bonferroni correction method (*α*'=0.004), there was no statistical significance in the nonlinear effects of age on diabetes awareness, treatment, and control rates, while other results remained unchanged. A statistical summary of the RCS models can be found in Supplementary Table S7.

## Discussion

This study assessed the prevalence, awareness, treatment, and control rates of hypertension, dyslipidemia, and diabetes in the southwestern plateau areas of China and identified various influencing factors. Our findings revealed a high prevalence of these three cardiometabolic disorders, but low rates of awareness, treatment, and control among the population. Notably, the rates of prevalence, awareness, treatment, and control of these diseases varied according to factors such as sex, altitude of residence, age, education level, income, current smoking and alcohol drinking behavior, and overweight/obesity. Altitude significantly modified the association between sociodemographic factors and cardiometabolic disorders.

The results of our study revealed high prevalence rates of cardiometabolic disorders in high-altitude areas: 46.20% for hypertension, 37.06% for dyslipidemia, and 11.79% for diabetes, exceeding national averages [hypertension 24.70% in 2018 (7), dyslipidemia 33.8% in 2019 (9), and diabetes 10.9% in 2013 (34)]. Possible factors contributing to these increases included improved diagnostic capabilities, population aging, and modifiable risk factors such as unhealthy lifestyles and overweight/obesity (7, 14, 35). Notably, awareness, treatment, and control rates were suboptimal, with hypertension rates (58.65%, 43.80%, 15.75%) comparable to national levels (43.3%, 38.7%, 12.9%), while dyslipidemia (15.90%, 5.77%, 2.11% vs. 11.7%, 10.1%, 4.8% in 2020–2022) and diabetes (27.54%, 21.68%, 6.86% vs. 36.7%, 32.9%, 50.1% in 2018) showed significantly lower treatment and control rates than national (36). These findings may be linked to the region's underdeveloped economic and insufficient healthcare systems. In the southwestern plateau areas of China, factors such as limited healthcare infrastructure, restricted access to essential medications, and transportation barriers collectively contribute to suboptimal disease management (37). This necessitates targeted interventions, such as enhanced health education, strengthened primary care capacity, and optimized healthcare resource allocation, to address the growing burden of cardiometabolic disorders in the region (38).

The prevalence, awareness, treatment, and control of hypertension, dyslipidemia, and diabetes were influenced by sex, age, education, income, smoking, alcohol use, and overweight/obesity (39, 40). Consistent with previous studies, males and older adults showed higher disease prevalence, though older individuals exhibited better awareness and control. Sex differences may be related to variations in hormones, lifestyle (diet, current smoking, alcohol consumption, fruit and vegetable consumption, etc.), and disparities in access to health information (18, 38). Females were more likely to be receptive to the concept of healthy living and had better performance in disease prevention behaviors, which helped them manage diseases more effectively (18). In contrast to some earlier findings (9, 41), higher education levels correlated with increased prevalences of dyslipidemia and diabetes, potentially due to social engagement patterns involving high-calorie diets and alcohol consumption (42). Our study found that higher income was associated with a higher dyslipidemia prevalence, but poorer control of hypertension. This may be explained by the fact that higher income leads to richer diets and more sedentary lifestyles, while limited access to healthcare and low awareness of standardized management in remote high-altitude areas. Consistent with previous studies, behavioral factors played a critical role in cardiometabolic disorders: smoking was consistently associated with poor disease control, alcohol use raised both incidence and impaired management (43), and overweight/obesity significantly increased the prevalence of all three conditions through physiological pathways such as elevated blood pressure (44), dyslipidemia (45), and insulin resistance (40). These findings highlight the need for targeted interventions addressing modifiable risks, especially smoking, alcohol, and obesity, while tailoring health communication to specific demographic groups to improve prevention and control of these diseases (35, 43).

The relationship between altitude and cardiometabolic disorders remains inconsistently reported. Some studies suggested a positive correlation between altitude and hypertension (15), others described U-shaped associations, with turning points near 3,800 meters (46). Several studies indicated that prolonged exposure to high altitude may increase the likelihood of dyslipidemia and lead to an increase in plasma glucose concentrations (43, 47). However, some studies reached opposite conclusions (48). Our study revealed that hypertension prevalence decreased with rising altitude and stabilized above 1,949 meters; dyslipidemia and diabetes prevalence decreased below 1,750 and 1,775 meters, respectively, peaked around 2,500 meters, and declined thereafter. Disease awareness, treatment, and control were optimal between 1,700–1,950 meters and diminished outside this range. Altitude also interacted with sociodemographic and behavioral factors, collectively shaping disease risk and management. These patterns may be explained by several mechanisms triggered by chronic hypoxia at high altitudes. This condition leads to increased hemoglobin concentration, which elevates blood viscosity and potentially raises peripheral vascular resistance (51). Simultaneously, it activates the sympathetic nervous system to increase heart rate and vasoconstriction, both of which contribute to the risk of hypertension. Persistent sympathetic activation disrupts glucose and lipid metabolism by promoting hepatic gluconeogenesis and lipolysis, which may explain the rise in dyslipidemia and diabetes around 2,500 meters (52). Additionally, lifestyle differences play a role; traditional highland diets rich in whole grains and dairy may mitigate hypertension and dyslipidemia risk but can impair insulin sensitivity, while low-altitude urban areas have more processed food intake and sedentary lifestyles, driving higher disease prevalence (16, 50). Furthermore, limited healthcare infrastructure, transportation barriers, and shortages of medical workers in mountainous regions above 2,500 meters reduce disease awareness and hinder treatment access, while the 1,700–1,950 meter range benefits from better healthcare coverage, aligning with optimal management outcomes (49). Genetic adaptations in long-term high-altitude residents enhance insulin sensitivity but may lead to compensatory hyperinsulinemia over time, further modulating disease risk (43, 47).

This study offers valuable insights for public health planning in the southwestern plateau areas of China. However, several limitations should be noted. First, participants with hypertension, dyslipidemia, and diabetes may be reluctant to report their diagnosis and medication use status, which may lead to an underestimation of these rates. Second, this study was conducted in several districts/counties in Yunnan Province, which may limit the representation of our findings and their applicability to other regions. Third, confounding factors such as dietary habits, physical activity, family history, and urban/rural status were not incorporated into the analysis, which may introduce bias in the results. Last, given the cross-sectional design, reverse causality cannot be discounted. Further mechanistic studies are needed to clarify the role of altitude in the development and management of cardiometabolic diseases.

## Conclusions

This study identified a high prevalence yet suboptimal control of cardiometabolic disorders (hypertension, diabetes, and dyslipidemia) in the southwestern plateau areas of China. High-risk populations included males, older adults, individuals with higher incomes, those who are overweight or obese, and alcohol drinkers. Treatment and control rates were significantly lower among residents living at altitudes ≥2,242 meters, younger adults, overweight or obese individuals, active smokers, and alcohol consumers. These findings underscore the urgent need for targeted public health strategies that prioritize these vulnerable groups and high-altitude regions through enhanced screening, accessible treatment, and tailored health education programs to improve the management and control of these diseases.

## Data Availability

The original contributions presented in the study are included in the article/Supplementary Material, further inquiries can be directed to the corresponding author.
