# Supplementary material for: Effects of altitude and sociodemographic factors on cardiometabolic disorders in the southwestern plateau areas of China
Source: Front Cardiovasc Med. 2026 May 15;13:1709651. doi: 10.3389/fcvm.2026.1709651 (PMC13218981; doi:10.3389/fcvm.2026.1709651)
Supplement: Supplementary file 1 [file Datasheet1.docx]

**Supplemental Materials for**

Effects of altitude and sociodemographic factors on cardiometabolic disorders in the southwestern plateau areas of China

Nan Zhang^1,2†^, Wenlong Zhu^1,2†^, Yu Xia^1^, Lin Duo^1,2^, Zhiling Luo^1,2^, Mingjing Tang^1,2*^, Da Zhu^1,2*^

^1^ Fuwai Yunnan Hospital, Chinese Academy of Medical Sciences, Affiliated Cardiovascular Hospital of Kunming Medical University, Kunming, China.

^2^ Yunnan Provincial Cardiovascular Clinical Medical Research Center, Kunming, China.

^†^ These authors contributed equally to this work.

***Corresponding author:**

Dr. Da Zhu, Fuwai Yunnan Hospital, Chinese Academy of Medical Sciences, Affiliated Cardiovascular Hospital of Kunming Medical University, Kunming 650102, China (Email: zhuda8687@126.com, telephone:+86-177-8073-1330).

Mingjing Tang, Fuwai Yunnan Hospital, Chinese Academy of Medical Sciences, Affiliated Cardiovascular Hospital of Kunming Medical University, Kunming 650102, China (Email: candysabrina@126.com, telephone:+86-151-0113-8758)

**Contents**

[Study design and participants 1](#_Toc225788010)

[Figure S1. Flowchart of on-site screening. 2](#_Toc225788011)

[Table S1. The association of altitude and sociodemographic factors with the rates of prevalence, awareness, treatment, and control of hypertension. 3](#_Toc225788012)

[Table S2. Subgroup analysis and interaction of different altitude and socio-demographic factors in prevalence, awareness, treatment, and control of hypertension. 4](#_Toc225788013)

[Table S3. The association of altitude and sociodemographic factors with the rates of prevalence, awareness, treatment, and control of dyslipidemia. 6](#_Toc225788014)

[Table S4. Subgroup analysis and interaction of different altitude and socio-demographic factors in prevalence, awareness, treatment, and control of dyslipidemia. 7](#_Toc225788015)

[Table S5. The association of altitude and sociodemographic factors with the rates of prevalence, awareness, treatment, and control of diabetes. 9](#_Toc225788016)

[Table S6. Subgroup analysis and interaction of different altitude and socio-demographic factors in prevalence, awareness, treatment, and control of diabetes. 10](#_Toc225788017)

[Table S7. Nonlinear relationship between age and altitude with the prevalence, awareness, treatment, and control of hypertension, dyslipidemia, and diabetes. 12](#_Toc225788018)

Study design and participants

The first investigation (China CVD-Risk Surveillance) utilized a stratified multi-stage random sampling design comprising three sequential phases in Yunnan Province. First, two communities and two townships were randomly selected from four urban districts and four rural counties, respectively. Subsequently, two committees/villages were randomly selected from each sampled community and township. Finally, 600 residents per committee/village (aged ≥18 years with ≥6 months' residency) underwent cardiovascular disease screening, yielding a total cohort of 9600 participants.^1^

In the second investigation (Cardiovascular Disease Risk Factors Among Plateau Area Residents of Yunnan Province), a multi-phase geographical stratification protocol was implemented. First, all townships in Yunnan Province situated above 2,500 m altitude were stratified into rural and urban strata, with two townships/streets per stratum selected via simple random sampling. Subsequently, four villages/committees were randomly sampled from each selected township/street, generating 16 sites. Finally, permanent residents aged ≥35 years with ≥6 months' residency in these townships/streets were enrolled as survey participants.^2^ The on-site screening and data collection processes of these two surveys are illustrated in **Figure S1**.

Reference

1. Pang L, Kottu L, Guo Z, et al. A tryst of 'blood pressure control- sex- comorbidities': the odyssey of basic public health services in Yunnan in quest for truth [J]. BMC Public Health, 2024,24(1): 490.
2. Pang L, Xia Y, Tang M, et al. Prevalence, spectrum and aetiology of valvular heart disease based on community echocardiographic screening transition from different altitudes in Yunnan, China [J]. Heart, 2025.

| **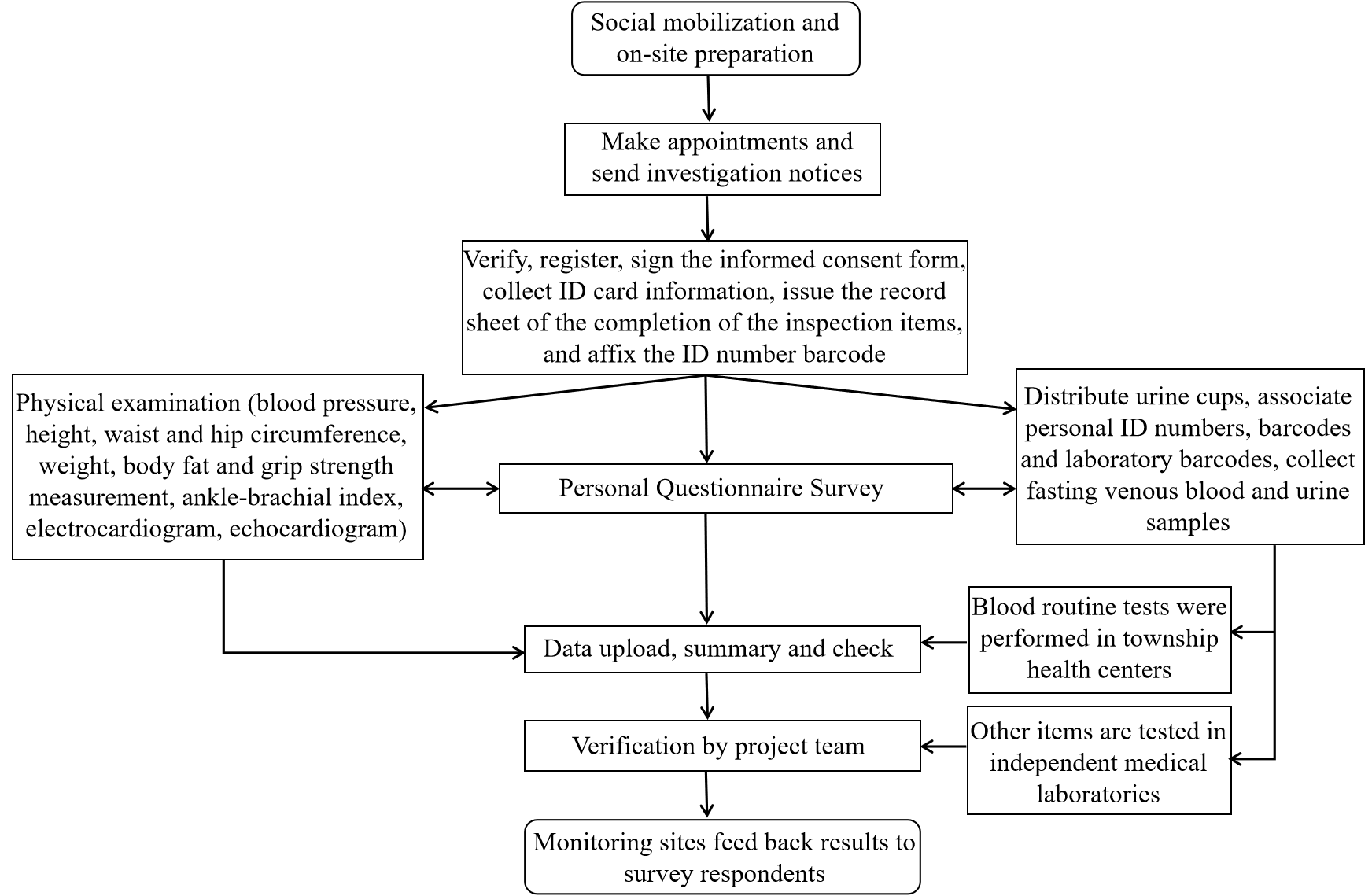** |
| --- |
| **Figure S1. Flowchart of on-site screening.** |

| Table S1. The association of altitude and sociodemographic factors with the rates of prevalence, awareness, treatment, and control of hypertension. | | | | | | | | | | | |
| --- | --- | --- | --- | --- | --- | --- | --- | --- | --- | --- | --- |
| **Characteristics** | **Prevalence** | |  | **Awareness** | |  | **Treatment** | |  | **Control** | |
|  | **OR (95% CI)** | ***p* value** |  | **OR (95% CI)** | ***p* value** |  | **OR (95% CI)** | ***p* value** |  | **OR (95% CI)** | ***p* value** |
| **Sex** |  |  |  |  |  |  |  |  |  |  |  |
| Female | 1.00 (Reference) | - |  | 1.00 (Reference) | - |  | 1.00 (Reference) | - |  | 1.00 (Reference) | - |
| Male | 1.31 (1.20 to 1.43) | <0.001 |  | 0.81 (0.71 to 0.93) | 0.002 |  | 0.73 (0.64 to 0.84) | <0.001 |  | 0.83 (0.70 to 1.00) | 0.049 |
| **Altitude groups, meters** |  |  |  |  |  |  |  |  |  |  |  |
| Altitude 1: [818, 1896) | 1.00 (Reference) | - |  | 1.00 (Reference) | - |  | 1.00 (Reference) | - |  | 1.00 (Reference) | - |
| Altitude 2: [1896, 2242) | 0.71 (0.63 to 0.79) | <0.001 |  | 1.02 (0.87 to 1.20) | 0.812 |  | 0.96 (0.82 to 1.13) | 0.607 |  | 0.93 (0.75 to 1.15) | 0.518 |
| Altitude 3: ≥2242 | 0.77 (0.69 to 0.86) | <0.001 |  | 0.92 (0.79 to 1.08) | 0.325 |  | 0.71 (0.60 to 0.83) | <0.001 |  | 0.81 (0.65 to 1.01) | 0.059 |
| Age groups, years |  |  |  |  |  |  |  |  |  |  |  |
| <60 | 1.00 (Reference) | - |  | 1.00 (Reference) | - |  | 1.00 (Reference) | - |  | 1.00 (Reference) | - |
| ≥60 | 3.41 (3.09 to 3.77) | <0.001 |  | 2.32 (2.02 to 2.66) | <0.001 |  | 2.40 (2.10 to 2.75) | <0.001 |  | 1.55 (1.29 to 1.86) | <0.001 |
| **Educational status** |  |  |  |  |  |  |  |  |  |  |  |
| Primary school and below | 1.00 (Reference) | - |  | 1.00 (Reference) | - |  | 1.00 (Reference) |  |  | 1.00 (Reference) | - |
| Junior high school and above | 0.67 (0.61 to 0.73) | <0.001 |  | 0.81 (0.71 to 0.93) | 0.003 |  | 0.76 (0.66 to 0.87) | <0.001 |  | 1.15 (0.95 to 1.38) | 0.146 |
| Income, yuan |  |  |  |  |  |  |  |  |  |  |  |
| <21000 | 1.00 (Reference) | - |  | 1.00 (Reference) | - |  | 1.00 (Reference) | - |  | 1.00 (Reference) | - |
| ≥21000 | 0.92 (0.84 to 1.01) | 0.075 |  | 0.90 (0.78 to 1.03) | 0.129 |  | 0.86 (0.75 to 0.98) | 0.028 |  | 0.81 (0.67 to 0.97) | 0.025 |
| **Current smoking** |  |  |  |  |  |  |  |  |  |  |  |
| No | 1.00 (Reference) | - |  | 1.00 (Reference) | - |  | 1.00 (Reference) | - |  | 1.00 (Reference) | - |
| Yes | 1.00 (0.90 to 1.10) | 0.924 |  | 0.67 (0.58 to 0.78) | <0.001 |  | 0.62 (0.53 to 0.72) | <0.001 |  | 0.68 (0.54 to 0.84) | <0.001 |
| **Current alcohol drinking** |  |  |  |  |  |  |  |  |  |  |  |
| No | 1.00 (Reference) | - |  | 1.00 (Reference) | - |  | 1.00 (Reference) | - |  | 1.00 (Reference) | - |
| Yes | 1.36 (1.22 to 1.53) | <0.001 |  | 0.58 (0.49 to 0.68) | <0.001 |  | 0.53 (0.45 to 0.63) | <0.001 |  | 0.50 (0.38 to 0.65) | <0.001 |
| **Overweight/obesity** |  |  |  |  |  |  |  |  |  |  |  |
| No | 1.00 (Reference) | - |  | 1.00 (Reference) | - |  | 1.00 (Reference) | - |  | 1.00 (Reference) | - |
| Yes | 1.98 (1.81 to 2.17) | <0.001 |  | 1.38 (1.21 to 1.58) | <0.001 |  | 1.31 (1.14 to 1.50) | <0.001 |  | 1.10 (0.92 to 1.32) | 0.308 |

| Table S2. Subgroup analysis and interaction of different altitude and socio-demographic factors in prevalence, awareness, treatment, and control of hypertension.^†^ | | | | | | | |
| --- | --- | --- | --- | --- | --- | --- | --- |
| **Characteristics^‡^** | **Altitude1** |  | **Altitude2** | |  | **Altitude3** | |
|  | **aOR (95% CI)** |  | **aOR (95% CI)** | ***p* for interaction** |  | **aOR (95% CI)** | ***p* for interaction** |
| **Prevalence** |  |  |  |  |  |  |  |
| Male | 1.40 (1.12 to 1.73)^*^ |  | 1.11 (0.88 to 1.39) | 0.004 |  | 1.39 (1.10 to 1.75)^*^ | 0.569 |
| ≥60 years | 4.35 (3.60 to 5.27)^*^ |  | 2.83 (2.32 to 3.47)^*^ | <0.001 |  | 3.64 (3.04 to 4.36)^*^ | 0.196 |
| Junior high school and above | 1.39 (1.18 to 1.64)^*^ |  | 0.79 (0.66 to 0.95)^*^ | <0.001 |  | 0.94 (0.77 to 1.16) | 0.026 |
| ≥21000 yuan | 0.62 (0.52 to 0.74)^*^ |  | 0.89 (0.73 to 1.08) | 0.066 |  | 0.90 (0.73 to 1.11) | 0.016 |
| Current smoking | 0.89 (0.71 to 1.12) |  | 0.77 (0.60 to 0.99)^*^ | 0.014 |  | 0.94 (0.73 to 1.20) | 0.507 |
| Current alcohol drinking | 1.56 (1.26 to 1.92)^*^ |  | 1.25 (0.98 to 1.60) | 0.024 |  | 1.51 (1.13 to 2.03)^*^ | 0.682 |
| Overweight/obesity | 2.35 (1.99 to 2.77)^*^ |  | 2.54 (2.14 to 3.03)^*^ | 0.220 |  | 2.06 (1.72 to 2.46)^*^ | 0.433 |
| **Awareness** |  |  |  |  |  |  |  |
| Male | 1.18 (0.88 to 1.58) |  | 0.68 (0.48 to 0.96)^*^ | 0.038 |  | 1.30 (0.94 to 1.82) | 0.857 |
| ≥60 years | 2.27 (1.79 to 2.88)^*^ |  | 2.29 (1.72 to 3.07)^*^ | 0.469 |  | 2.81 (2.17 to 3.65)^*^ | 0.307 |
| Junior high school and above | 0.69 (0.55 to 0.86)^*^ |  | 1.40 (1.05 to 1.87)^*^ | <0.001 |  | 1.11 (0.82 to 1.51) | 0.007 |
| ≥21000 yuan | 0.99 (0.78 to 1.26) |  | 0.98 (0.73 to 1.32) | 0.735 |  | 1.14 (0.83 to 1.56) | 0.307 |
| Current smoking | 0.83 (0.61 to 1.12) |  | 1.24 (0.86 to 1.80) | 0.863 |  | 0.65 (0.46 to 0.93)^*^ | 0.510 |
| Current alcohol drinking | 0.66 (0.50 to 0.88)^*^ |  | 0.50 (0.35 to 0.73)^*^ | 0.095 |  | 0.72 (0.49 to 1.07) | 0.803 |
| Overweight/obesity | 1.47 (1.18 to 1.85)^*^ |  | 1.81 (1.38 to 2.38)^*^ | 0.252 |  | 1.57 (1.21 to 2.04)^*^ | 0.742 |
| **Treatment** |  |  |  |  |  |  |  |
| Male | 1.03 (0.77 to 1.37) |  | 0.62 (0.44 to 0.86)^*^ | 0.142 |  | 1.23 (0.89 to 1.68) | 0.116 |
| ≥60 years | 2.80 (2.22 to 3.54)^*^ |  | 2.82 (2.13 to 3.74)^*^ | 0.474 |  | 1.85 (1.43 to 2.40)^*^ | 0.009 |
| Junior high school and above | 0.76 (0.61 to 0.95)^*^ |  | 1.16 (0.87 to 1.55) | 0.029 |  | 0.84 (0.62 to 1.13) | 0.429 |
| ≥21000 yuan | 0.97 (0.76 to 1.24) |  | 0.98 (0.73 to 1.32) | 0.764 |  | 1.15 (0.84 to 1.57) | 0.112 |
| Current smoking | 0.78 (0.57 to 1.06) |  | 1.12 (0.76 to 1.63) | 0.795 |  | 0.79 (0.56 to 1.13) | 0.204 |
| Current alcohol drinking | 1.03 (0.77 to 1.37) |  | 0.60 (0.40 to 0.88)^*^ | 0.886 |  | 0.70 (0.46 to 1.04) | 0.186 |
| Overweight/obesity | 1.36 (1.09 to 1.72)^*^ |  | 1.80 (1.36 to 2.38)^*^ | 0.121 |  | 1.36 (1.05 to 1.77)^*^ | 0.602 |
| **Control** |  |  |  |  |  |  |  |
| Male | 1.09 (0.76 to 1.55) |  | 0.69 (0.44 to 1.08) | 0.099 |  | 1.36 (0.89 to 2.07) | 0.603 |
| ≥60 years | 1.72 (1.27 to 2.34)^*^ |  | 1.63 (1.13 to 2.36)^*^ | 0.507 |  | 1.41 (0.98 to 2.03) | 0.427 |
| Junior high school and above | 0.51 (0.38 to 0.68)^*^ |  | 1.28 (0.88 to 1.85) | <0.001 |  | 0.88 (0.57 to 1.32) | 0.027 |
| ≥21000 yuan | 1.48 (1.07 to 2.03)^*^ |  | 1.38 (0.93 to 2.06) | 0.815 |  | 1.57 (1.04 to 2.34)^*^ | 0.485 |
| Current smoking | 0.76 (0.50 to 1.14) |  | 1.10 (0.65 to 1.89) | 0.778 |  | 0.74 (0.45 to 1.19) | 0.874 |
| Current alcohol drinking | 0.63 (0.43 to 0.92)^*^ |  | 0.35 (0.18 to 0.66)^*^ | 0.095 |  | 0.33 (0.14 to 0.66)^*^ | 0.212 |
| Overweight/obesity | 1.18 (0.88 to 1.59) |  | 1.18 (0.82 to 1.71) | 0.945 |  | 1.02 (0.71 to 1.46) | 0.729 |
| † Altitude 1: [818, 1896) meters, Altitude 2: [1896, 2242) meters, Altitude 3: ≥2242 meters. The model was adjusted for sex, age, education status, income, overweight/obesity, current smoking, and current alcohol drinking status.  **‡** Reference level was female, <60 years, primary school and below, <21000 yuan, normal weight, non-smoker, non-alcohol drinker.  * indicates a statistical difference (*p*<0.05) compared to the reference group. | | | | | | | |

| Table S3. The association of altitude and sociodemographic factors with the rates of prevalence, awareness, treatment, and control of dyslipidemia. | | | | | | | | | | | |
| --- | --- | --- | --- | --- | --- | --- | --- | --- | --- | --- | --- |
| **Characteristics** | **Prevalence** | |  | **Awareness** | |  | **Treatment** | |  | **Control** | |
|  | **OR (95% CI)** | ***p* value** |  | **OR (95% CI)** | ***p* value** |  | **OR (95% CI)** | ***p* value** |  | **OR (95% CI)** | ***p* value** |
| **Sex** |  |  |  |  |  |  |  |  |  |  |  |
| Female | 1.00 (Reference) | - |  | 1.00 (Reference) | - |  | 1.00 (Reference) | - |  | 1.00 (Reference) | - |
| Male | 1.57 (1.43 to 1.73) | <0.001 |  | 1.00 (0.81 to 1.22) | 0.967 |  | 0.90 (0.66 to 1.24) | 0.514 |  | 0.90 (0.54 to 1.50) | 0.680 |
| **Altitude groups, meters^*^** |  |  |  |  |  |  |  |  |  |  |  |
| Altitude 1: [818, 1896) | 1.00 (Reference) | - |  | 1.00 (Reference) | - |  | 1.00 (Reference) | - |  | 1.00 (Reference) | - |
| Altitude 2: [1896, 2242) | 0.93 (0.83 to 1.04) | 0.204 |  | 1.04 (0.84 to 1.30) | 0.700 |  | 0.66 (0.48 to 0.91) | 0.011 |  | 0.40 (0.24 to 0.68) | <0.001 |
| Altitude 3: ≥2242 | 0.67 (0.60 to 0.75) | <0.001 |  | 0.36 (0.26 to 0.48) | <0.001 |  |  |  |  |  |  |
| Age groups, years |  |  |  |  |  |  |  |  |  |  |  |
| <60 | 1.00 (Reference) | - |  | 1.00 (Reference) | - |  | 1.00 (Reference) | - |  | 1.00 (Reference) | - |
| ≥60 | 1.11 (1.01 to 1.22) | 0.037 |  | 1.15 (0.93 to 1.42) | 0.180 |  | 1.86 (1.35 to 2.55) | <0.001 |  | 2.76 (1.65 to 4.68) | <0.001 |
| **Educational status** |  |  |  |  |  |  |  |  |  |  |  |
| Primary school and below | 1.00 (Reference) | - |  | 1.00 (Reference) | - |  | 1.00 (Reference) | - |  | 1.00 (Reference) | - |
| Junior high school and above | 1.25 (1.14 to 1.38) | <0.001 |  | 1.80 (1.47 to 2.21) | <0.001 |  | 1.46 (1.06 to 2.01) | 0.020 |  | 2.33 (1.37 to 4.08) | 0.002 |
| Income, yuan |  |  |  |  |  |  |  |  |  |  |  |
| <21000 | 1.00 (Reference) | - |  | 1.00 (Reference) | - |  | 1.00 (Reference) | - |  | 1.00 (Reference) | - |
| ≥21000 | 1.24 (1.13 to 1.36) | <0.001 |  | 1.43 (1.17 to 1.75) | <0.001 |  | 1.16 (0.85 to 1.59) | 0.352 |  | 1.80 (1.08 to 3.05) | 0.026 |
| **Current smoking** |  |  |  |  |  |  |  |  |  |  |  |
| No | 1.00 (Reference) | - |  | 1.00 (Reference) | - |  | 1.00 (Reference) | - |  | 1.00 (Reference) | - |
| Yes | 1.38 (1.24 to 1.53) | <0.001 |  | 0.80 (0.64 to 1.00) | 0.051 |  | 0.61 (0.41 to 0.88) | 0.009 |  | 0.59 (0.30 to 1.06) | 0.092 |
| **Current alcohol drinking** |  |  |  |  |  |  |  |  |  |  |  |
| No | 1.00 (Reference) | - |  | 1.00 (Reference) | - |  | 1.00 (Reference) | - |  | 1.00 (Reference) | - |
| Yes | 1.47 (1.31 to 1.65) | <0.001 |  | 0.98 (0.77 to 1.24) | 0.886 |  | 0.67 (0.44 to 1.01) | 0.063 |  | 0.83 (0.42 to 1.52) | 0.572 |
| **Overweight/obesity** |  |  |  |  |  |  |  |  |  |  |  |
| No | 1.00 (Reference) | - |  | 1.00 (Reference) | - |  | 1.00 (Reference) | - |  | 1.00 (Reference) | - |
| Yes | 2.25 (2.05 to 2.47) | <0.001 |  | 1.66 (1.34 to 2.07) | <0.001 |  | 1.24 (0.89 to 1.74) | 0.207 |  | 0.67 (0.40 to 1.13) | 0.128 |
| ***** In the analysis of dyslipidemia treatment and control rate, Altitude 2 and 3 were merged into Altitude 2 (≥ 1896 meters). | | | | | | | | | | | |

| Table S4. Subgroup analysis and interaction of different altitude and socio-demographic factors in prevalence, awareness, treatment, and control of dyslipidemia.^†^ | | | | | | | |
| --- | --- | --- | --- | --- | --- | --- | --- |
| **Characteristics^‡^** | **Altitude1** |  | **Altitude2** | |  | **Altitude3** | |
|  | **aOR(95% CI)** |  | **aOR(95% CI)** | ***p* for interaction** |  | **aOR(95% CI)** | ***p* for interaction** |
| **Prevalence** |  |  |  |  |  |  |  |
| Male | 1.48 (1.20 to 1.82)^*^ |  | 1.36 (1.08 to 1.70)^*^ | 0.369 |  | 1.22 (0.96 to 1.53) | 0.379 |
| ≥60 years | 1.20 (1.01 to 1.44)^*^ |  | 1.23 (1.01 to 1.51)^*^ | 0.845 |  | 1.24 (1.03 to 1.49)^*^ | 0.767 |
| Junior high school and above | 1.33 (1.13 to 1.56)^*^ |  | 1.16 (0.96 to 1.39) | 0.260 |  | 0.88 (0.71 to 1.08) | 0.007 |
| ≥21000 yuan | 0.95 (0.80 to 1.12) |  | 1.04 (0.85 to 1.26) | 0.854 |  | 1.51 (1.23 to 1.86)^*^ | 0.014 |
| Current smoking | 1.20 (0.97 to 1.50) |  | 1.09 (0.86 to 1.39) | 0.325 |  | 1.04 (0.81 to 1.33) | 0.365 |
| Current alcohol drinking | 1.05 (0.86 to 1.29) |  | 1.05 (0.82 to 1.33) | 0.545 |  | 1.47 (1.10 to 1.95)^*^ | 0.125 |
| Overweight/obesity | 2.65 (2.26 to 3.11)^*^ |  | 2.43 (2.05 to 2.89)^*^ | 0.564 |  | 1.65 (1.38 to 1.97)^*^ | <0.001 |
| **Awareness** |  |  |  |  |  |  |  |
| Male | 1.10 (0.73 to 1.64) |  | 0.86 (0.56 to 1.29) | 0.074 |  | 0.89 (0.44 to 1.76) | 0.324 |
| ≥60 years | 2.02 (1.44 to 2.85)^*^ |  | 1.19 (0.81 to 1.74) | 0.158 |  | 0.72 (0.39 to 1.30) | 0.004 |
| Junior high school and above | 1.07 (0.78 to 1.47) |  | 1.06 (0.74 to 1.51) | 0.788 |  | 1.90 (1.04 to 3.43)^*^ | 0.047 |
| ≥21000 yuan | 2.02 (1.43 to 2.87)^*^ |  | 1.47 (1.00 to 2.17) | 0.224 |  | 2.46 (1.34 to 4.51)^*^ | 0.399 |
| Current smoking | 0.91 (0.61 to 1.35) |  | 0.70 (0.44 to 1.12) | 0.115 |  | 0.48 (0.21 to 1.06) | 0.164 |
| Current alcohol drinking | 0.97 (0.66 to 1.41) |  | 0.92 (0.56 to 1.48) | 0.417 |  | 0.62 (0.20 to 1.56) | 0.346 |
| Overweight/obesity | 1.25 (0.91 to 1.75) |  | 1.58 (1.10 to 2.30)^*^ | 0.301 |  | 2.19 (1.24 to 4.01)^*^ | 0.040 |
| **Treatment** |  |  |  |  |  |  |  |
| Male | 1.50 (0.84 to 2.67) |  | 0.83 (0.47 to 1.43) | 0.069 |  | - | - |
| ≥60 years | 3.12 (1.89 to 5.19)^*^ |  | 1.45 (0.90 to 2.33) | 0.035 |  | - | - |
| Junior high school and above | 0.97 (0.60 to 1.56) |  | 1.11 (0.68 to 1.80) | 0.615 |  | - | - |
| ≥21000 yuan | 2.01 (1.20 to 3.42)^*^ |  | 1.81 (1.10 to 2.99)^*^ | 0.864 |  | - | - |
| Current smoking | 0.61 (0.33 to 1.11) |  | 0.66 (0.33 to 1.28) | 0.427 |  | - | - |
| Current alcohol drinking | 0.85 (0.47 to 1.52) |  | 0.45 (0.17 to 1.01) | 0.156 |  | - | - |
| Overweight/obesity | 0.90 (0.56 to 1.46) |  | 1.74 (1.09 to 2.88)^*^ | 0.046 |  | - | - |
| **Control** |  |  |  |  |  |  |  |
| Male | 1.09 (0.47 to 2.49) |  | 0.67 (0.22 to 1.83) | 0.669 |  | - | - |
| ≥60 years | 4.16 (2.00 to 8.94)^*^ |  | 3.29 (1.36 to 8.04)^*^ | 0.322 |  | - | - |
| Junior high school and above | 0.89 (0.45 to 1.78) |  | 2.98 (1.21 to 7.85)^*^ | 0.020 |  | - | - |
| ≥21000 yuan | 3.02 (1.42 to 6.64)^*^ |  | 4.03 (1.51 to 12.11)^*^ | 0.351 |  | - | - |
| Current smoking | 0.50 (0.19 to 1.25) |  | 0.86 (0.24 to 2.97) | 0.758 |  | - | - |
| Current alcohol drinking | 1.10 (0.45 to 2.58) |  | 0.51 (0.08 to 1.99) | 0.504 |  | - | - |
| Overweight/obesity | 0.52 (0.26 to 1.01) |  | 0.96 (0.41 to 2.30) | 0.234 |  | - | - |
| † Altitude 1: [818, 1896) meters, Altitude 2: [1896, 2242) meters, Altitude 3: ≥2242 meters. In the analysis of dyslipidemia treatment and control rate, Altitude 2 and 3 were merged into Altitude 2 (≥ 1896 meters). The model was adjusted for sex, age, education status, income, overweight/obesity, current smoking, and current alcohol drinking status.  **‡** Reference level was female, <60 years, primary school and below, <21000 yuan, normal weight, non-smoker, non-alcohol drinker.  * indicates a statistical difference compared to the reference group. | | | | | | | |

| Table S5. The association of altitude and sociodemographic factors with the rates of prevalence, awareness, treatment, and control of diabetes. | | | | | | | | | | | |
| --- | --- | --- | --- | --- | --- | --- | --- | --- | --- | --- | --- |
| **Characteristics** | **Prevalence** | |  | **Awareness** | |  | **Treatment** | |  | **Control** | |
|  | **OR (95% CI)** | ***p* value** |  | **OR (95% CI)** | ***p* value** |  | **OR (95% CI)** | ***p* value** |  | **OR (95% CI)** | ***p* value** |
| **Sex** |  |  |  |  |  |  |  |  |  |  |  |
| Female | 1.00 (Reference) | - |  | 1.00 (Reference) | - |  | 1.00 (Reference) | - |  | 1.00 (Reference) | - |
| Male | 1.13 (0.98 to 1.30) | 0.083 |  | 1.16 (0.87 to 1.56) | 0.313 |  | 1.03 (0.75 to 1.42) | 0.844 |  | 0.87 (0.52 to 1.47) | 0.611 |
| **Altitude groups, meters^*^** |  |  |  |  |  |  |  |  |  |  |  |
| Altitude 1: [818, 1896) | 1.00 (Reference) | - |  | 1.00 (Reference) | - |  | 1.00 (Reference) | - |  | 1.00 (Reference) | - |
| Altitude 2: [1896, 2242) | 0.95 (0.79 to 1.14) | 0.591 |  | 0.22 (0.16 to 0.29) | <0.001 |  | 0.27 (0.19 to 0.37) | <0.001 |  | 0.30 (0.17 to 0.50) | <0.001 |
| Altitude 3: ≥2242 | 1.95 (1.65 to 2.30) | <0.001 |  |  |  |  |  |  |  |  |  |
| Age groups, years |  |  |  |  |  |  |  |  |  |  |  |
| <60 | 1.00 (Reference) | - |  | 1.00 (Reference) | - |  | 1.00 (Reference) | - |  | 1.00 (Reference) | - |
| ≥60 | 1.78 (1.54 to 2.05) | <0.001 |  | 1.83 (1.36 to 2.46) | <0.001 |  | 1.72 (1.25 to 2.37) | <0.001 |  | 2.40 (1.41 to 4.17) | 0.001 |
| **Educational status** |  |  |  |  |  |  |  |  |  |  |  |
| Primary school and below | 1.00 (Reference) | - |  | 1.00 (Reference) | - |  | 1.00 (Reference) | - |  | 1.00 (Reference) | - |
| Junior high school and above | 0.73 (0.63 to 0.84) | <0.001 |  | 1.36 (1.01 to 1.83) | 0.043 |  | 1.16 (0.84 to 1.61) | 0.361 |  | 0.75 (0.42 to 1.30) | 0.322 |
| Income, yuan |  |  |  |  |  |  |  |  |  |  |  |
| <21000 | 1.00 (Reference) | - |  | 1.00 (Reference) | - |  | 1.00 (Reference) | - |  | 1.00 (Reference) | - |
| ≥21000 | 0.77 (0.66 to 0.89) | <0.001 |  | 1.47 (1.09 to 1.98) | 0.012 |  | 1.32 (0.95 to 1.82) | 0.097 |  | 1.17 (0.68 to 1.98) | 0.559 |
| **Current smoking** |  |  |  |  |  |  |  |  |  |  |  |
| No | 1.00 (Reference) | - |  | 1.00 (Reference) | - |  | 1.00 (Reference) | - |  | 1.00 (Reference) | - |
| Yes | 0.97 (0.83 to 1.13) | 0.721 |  | 0.93 (0.66 to 1.28) | 0.644 |  | 0.81 (0.55 to 1.16) | 0.251 |  | 0.62 (0.31 to 1.15) | 0.153 |
| **Current alcohol drinking** |  |  |  |  |  |  |  |  |  |  |  |
| No | 1.00 (Reference) | - |  | 1.00 (Reference) | - |  | 1.00 (Reference) | - |  | 1.00 (Reference) | - |
| Yes | 0.96 (0.80 to 1.15) | 0.683 |  | 1.02 (0.69 to 1.47) | 0.931 |  | 1.02 (0.67 to 1.51) | 0.941 |  | 0.72 (0.33 to 1.43) | 0.384 |
| **Overweight/obesity** |  |  |  |  |  |  |  |  |  |  |  |
| No | 1.00 (Reference) | - |  | 1.00 (Reference) | - |  | 1.00 (Reference) | - |  | 1.00 (Reference) | - |
| Yes | 1.37 (1.19 to 1.58) | <0.001 |  | 2.20 (1.62 to 3.01) | <0.001 |  | 2.18 (1.56 to 3.08) | <0.001 |  | 1.61 (0.95 to 2.83) | 0.084 |
| * In the analysis of diabetes awareness, treatment, and control, Altitude 2 and 3 were merged into Altitude 2 (≥ 1896 meters). | | | | | | | | | | | |

| Table S6. Subgroup analysis and interaction of different altitude and socio-demographic factors in prevalence, awareness, treatment, and control of diabetes.^†^ | | | | | | | |
| --- | --- | --- | --- | --- | --- | --- | --- |
| **Characteristics^‡^** | **Altitude1** |  | **Altitude2** | |  | **Altitude3** | |
|  | **aOR(95% CI)** |  | **aOR(95% CI)** | ***p* for interaction** |  | **aOR(95% CI)** | ***p* for interaction** |
| **Prevalence** |  |  |  |  |  |  |  |
| Male | 1.49 (1.07 to 2.06)^*^ |  | 1.29 (0.89 to 1.85) | 0.931 |  | 0.93 (0.70 to 1.23) | 0.230 |
| ≥60 years | 2.51 (1.91 to 3.31)^*^ |  | 1.66 (1.22 to 2.26)^*^ | 0.065 |  | 1.25 (1.00 to 1.56)^*^ | <0.001 |
| Junior high school and above | 1.10 (0.85 to 1.43) |  | 0.75 (0.54 to 1.03) | 0.061 |  | 0.97 (0.75 to 1.24) | 0.944 |
| ≥21000 yuan | 0.79 (0.59 to 1.05) |  | 0.83 (0.60 to 1.14) | 0.932 |  | 1.49 (1.16 to 1.91)^*^ | <0.001 |
| Current smoking | 0.78 (0.55 to 1.10) |  | 1.09 (0.74 to 1.62) | 0.260 |  | 0.90 (0.66 to 1.24) | 0.637 |
| Current alcohol drinking | 1.09 (0.79 to 1.51) |  | 0.87 (0.57 to 1.30) | 0.616 |  | 1.48 (1.04 to 2.07)^*^ | 0.183 |
| Overweight/obesity | 1.90 (1.46 to 2.48)^*^ |  | 1.96 (1.46 to 2.66)^*^ | 0.690 |  | 1.19 (0.95 to 1.48) | 0.026 |
| **Awareness** |  |  |  |  |  |  |  |
| Male | 0.99 (0.54 to 1.82) |  | 1.08 (0.62 to 1.87) | 0.333 |  | - | - |
| ≥60 years | 1.82 (1.09 to 3.05)^*^ |  | 1.83 (1.19 to 2.84)^*^ | 0.430 |  | - | - |
| Junior high school and above | 0.68 (0.41 to 1.11) |  | 1.34 (0.83 to 2.13) | 0.016 |  | - | - |
| ≥21000 yuan | 1.11 (0.65 to 1.89) |  | 1.58 (0.99 to 2.51) | 0.086 |  | - | - |
| Current smoking | 1.02 (0.51 to 2.05) |  | 1.11 (0.62 to 2.01) | 0.620 |  | - | - |
| Current alcohol drinking | 0.65 (0.35 to 1.21) |  | 0.78 (0.39 to 1.46) | 0.342 |  | - | - |
| Overweight/obesity | 1.32 (0.78 to 2.23) |  | 3.01 (1.93 to 4.81)^*^ | 0.012 |  | - | - |
| **Treatment** |  |  |  |  |  |  |  |
| Male | 1.02 (0.55 to 1.89) |  | 0.92 (0.50 to 1.68) | 0.254 |  | - | - |
| ≥60 years | 1.72 (1.02 to 2.93)^*^ |  | 1.56 (0.97 to 2.49) | 0.251 |  | - | - |
| Junior high school and above | 0.65 (0.39 to 1.09) |  | 1.30 (0.77 to 2.15) | 0.024 |  | - | - |
| ≥21000 yuan | 0.99 (0.57 to 1.70) |  | 1.27 (0.76 to 2.10) | 0.146 |  | - | - |
| Current smoking | 0.61 (0.29 to 1.25) |  | 1.24 (0.65 to 2.38) | 0.084 |  | - | - |
| Current alcohol drinking | 0.83 (0.43 to 1.59) |  | 0.94 (0.45 to 1.83) | 0.294 |  | - | - |
| Overweight/obesity | 1.25 (0.73 to 2.15) |  | 3.07 (1.88 to 5.15)^*^ | 0.012 |  | - | - |
| **Control** |  |  |  |  |  |  |  |
| Male | 1.47 (0.62 to 3.45) |  | 0.67 (0.20 to 1.85) | 0.426 |  | - | - |
| ≥60 years | 2.64 (1.19 to 6.36)^*^ |  | 1.59 (0.72 to 3.56) | 0.301 |  | - | - |
| Junior high school and above | 0.71 (0.33 to 1.48) |  | 1.50 (0.60 to 3.49) | 0.188 |  | - | - |
| ≥21000 yuan | 0.71 (0.30 to 1.57) |  | 0.81 (0.31 to 1.96) | 0.742 |  | - | - |
| Current smoking | 0.66 (0.21 to 1.93) |  | 1.16 (0.33 to 4.18) | 0.865 |  | - | - |
| Current alcohol drinking | 0.70 (0.25 to 1.82) |  | 0.59 (0.09 to 2.22) | 0.807 |  | - | - |
| Overweight/obesity | 1.45 (0.67 to 3.31) |  | 1.65 (0.75 to 3.82) | 0.718 |  | - | - |
| † Altitude 1: [818, 1896) meters, Altitude 2: [1896, 2242) meters, Altitude 3: ≥2242 meters. In the analysis of diabetes awareness, treatment, and control, Altitude 2 and 3 were merged into Altitude 2 (≥ 1896 meters). The model was adjusted for sex, age, education status, income, overweight/obesity, current smoking, and current alcohol drinking status.  ‡ Reference level was female, <60 years, primary school and below, <21000 yuan, normal weight, non-smoker, non-alcohol drinker.  * indicates a statistical difference compared to the reference group. | | | | | | | |

Table S7. Nonlinear relationship between age and altitude with the prevalence, awareness, treatment, and control of hypertension, dyslipidemia, and diabetes.

| **Diseases, rates** | **Age** | | **Nonlinear** | |  | **Altitude** | | **Nonlinear** | |
| --- | --- | --- | --- | --- | --- | --- | --- | --- | --- |
|  | **χ2** | ***p* value** | **χ2** | ***p* value** |  | **χ2** | ***p* value** | **χ2** | ***p* value** |
| **Hypertension** |  |  |  |  |  |  |  |  |  |
| Prevalence | 836.25 | <0.001 | 44.61 | <0.001 |  | 30.44 | <0.001 | 21.11 | <0.001 |
| Awareness | 206.84 | <0.001 | 34.67 | <0.001 |  | 11.37 | 0.011 | 11.16 | 0.004 |
| Treatment | 236.93 | <0.001 | 37.91 | <0.001 |  | 28.74 | <0.001 | 11.96 | 0.003 |
| Control | 52.54 | <0.001 | 26.01 | <0.001 |  | 7.70 | 0.059 | 4.03 | 0.101 |
| **Dyslipidemia** |  |  |  |  |  |  |  |  |  |
| Prevalence | 53.59 | <0.001 | 20.55 | <0.001 |  | 61.27 | <0.001 | 42.93 | <0.001 |
| Awareness | 42.80 | <0.001 | 15.22 | <0.001 |  | 32.58 | <0.001 | 19.14 | <0.001 |
| Treatment | 35.18 | <0.001 | 11.07 | 0.004 |  | 9.78 | 0.020 | 4.08 | 0.130 |
| Control | 28.37 | <0.001 | 2.45 | 0.294 |  | 10.56 | 0.014 | 5.68 | 0.058 |
| **Diabetes** |  |  |  |  |  |  |  |  |  |
| Prevalence | 99.44 | <0.001 | 30.02 | <0.001 |  | 110.31 | <0.001 | 70.97 | <0.001 |
| Awareness | 26.21 | <0.001 | 7.64 | 0.022 |  | 117.49 | <0.001 | 64.18 | <0.001 |
| Treatment | 16.06 | 0.001 | 6.22 | 0.045 |  | 86.93 | <0.001 | 44.29 | <0.001 |
| Control | 10.48 | 0.015 | 6.95 | 0.032 |  | 25.62 | <0.001 | 13.35 | 0.001 |
